# Long-term outcomes of infantile spasms in children treated with ketogenic diet therapy in combination with anti-seizure medications in a resource-limited region

**DOI:** 10.3389/fepid.2022.1080068

**Published:** 2023-01-16

**Authors:** Jianxiang Liao, Zhanqi Hu, Sufang Lin, Xinguo Lu, Jialun Wen, Jing Duan, Dongfang Zou, Huafang Zou, Mei Yu, Liqin Liu, Xiaoying Qiao, Yuanzhen Ye

**Affiliations:** Shenzhen Children's Hospital, Shenzhen, China

**Keywords:** epilepsy, infantile spasms, anti-seizure medications, seizure free, ketogenic diet, outcome, treatment, real world study

## Abstract

**Objective:**

Despite numerous guidelines, the overall outcome of infantile spasms is poor, with only a small number of patients being able to attend school. The purpose of this study was to investigate long-term outcomes. Patients had poor access to the recommended first-line anti-seizure medications (ASMs), such as hormones (corticotropin or prednisolone/prednisone) and vigabatrin, and their alternative treatment was other ASMs and a ketogenic diet.

**Methods:**

Patients suffering from infantile spasms who had at least 2 years of medical records in the electronic medical record system between January 2014 and August 2022 were included in this study. Patient information was retrospectively reviewed. All patients had received ketogenic diet therapy (mainly classical ketogenic diet therapy). The ketogenic diet therapy was combined with ASMs not used as first-line therapies. The primary endpoint outcome measure was the number of patients with seizure freedom. The secondary measures included the duration of ketogenic diet therapy, choice of ASMs, and patient development at the last visit.

**Results:**

A total of 177 patients with infantile spasms were included, and 152 (86%) of them had seizure freedom. The median duration from the first to the last hospital visit was 53.27 months, and the number of visits was 47.00. The median age at the initial hospital visit was 8.00 months, and the median age at initiation of the ketogenic diet was 17.73 months. At the last visit, the proportions of patients with neurodevelopmental delay, developmental epileptic encephalopathy, drug-resistant epilepsy, and generalized seizures increased significantly. The frequently used ASMs were topiramate, valproic acid, levetiracetam, nitrazepam, and vitamin B6 injection, while the recommended first-line drugs corticotropin and vigabatrin were rarely selected. The study duration of 9.5 years was divided into three periods but the prescription of ASMs did not change significantly between these periods.

**Conclusions:**

Although the seizure freedom rate was high with ketogenic diet therapy combined with non-standard ASMs, the patients had a significant neurodevelopmental delay at the last visit, which was, however, similar to that of standard treatment. To improve the outcomes of infantile spasms, multicenter clinical trials of the ketogenic diet as a first-line treatment in combination with non-standard ASMs are needed.

## Introduction

Infantile spasms are a common epilepsy syndrome in children with diverse etiologies, and although several treatment options are available, the long-term outcome is mostly poor (1–3). In a retrospective study, only 42 out of 127 (33%) patients had favorable treatment outcomes at the last follow-up (4). A retrospective record review reported that the second and third treatments are started within 14 days after treatment failure in only 21% and 24% of newly diagnosed spasms, respectively (5). Only 15% of infantile spasms of unknown causes have normal development, while 85% of patients have a developmental delay at the last assessment (median 2.7 years); therefore, they are classified into catastrophic epilepsy (4, 6). The pathogenesis of infantile spasms remains unclear and thus there are limited therapeutic approaches for the disease (7, 8).

Ketogenic diet (KD) is a special diet that is composed of high proportions of fat, low proportions of carbohydrate, and appropriate proportions of proteins and other nutrients (9, 10). Since its introduction over 100 years ago, the ketogenic diet remains one of the main choices for drug-resistant epilepsy treatment and has shown neuroprotective effects in improving cognition and enabling normal social activities (11, 12). Numerous guidelines recommend two agents for the treatment of infantile spasms: hormones (corticotropin or prednisolone/prednisone) and vigabatrin (a standard treatment). Their use as first-line agents could control epileptic spasms as early as possible, potentially improving neurodevelopmental outcomes. However, there is a limited supply of these two first-line drugs in mainland China. The hormones are natural adrenocorticotropic hormones extracted from the pituitary glands of animals in China, and they are used in less than 50% of children with infantile spasms due to the limited production due to their low price. Vigabatrin was not officially listed and made available as an ASM in mainland China until 2022. There are few reports on the real-world epidemiology of treatment in China. In this study, we retrospectively reviewed the treatment and outcomes of infantile spasms in 177 patients who received ketogenic diet therapy at our institution to understand the real-world treatment landscape in resource-limited settings, discuss possible alternative strategies, highlight the need for first-line treatments of this disease, and call for further research to find better treatments to improve the long-term outcome of the disease.

## Materials and methods

### Patients and methods

Patients with a diagnosis of infantile spasms or West syndrome, according to the International League Against Epilepsy diagnostic criteria for infantile spasms (infantile epileptic spasm syndrome) (13–15), were included in this study. All patients had an age of disease onset younger than 2 years, were treated with a ketogenic diet (mainly a classical ketogenic diet therapy), had medical information available in the electronic medical record system for more than 2 years, and had multiple visits to hospital from January 2014 to August 2022. Data were collected from the Comprehensive Pediatric Epilepsy Center, Shenzhen Children's Hospital. The study was a retrospective case note review. The primary endpoint outcome was the number of patients achieving seizure freedom. The cessation of spasms was defined as no witnessed spasms as recorded by a pediatric neurologist in the electronic medical record system. The pediatric neurologist mainly determined the response to treatment using seizure diaries and oral reports when patients visited our hospital. Secondary outcomes included the sequence of the ketogenic diet as anti-seizure treatment was introduced and its combination with drug treatment, and the neurodevelopmental status of the patients at the last visit. This study was approved by the Shenzhen Children's Hospital Ethics Committee [approval ID: 2015(012), clinical trial registration number, ChiCTR-IIR-16008342].

### Definition and medical records

Medical records included outpatient medical records, inpatient medical records, admission records, prescription records, and discharge records. Seizure freedom was designated if medical records documented seizure control to achieve seizure freedom. The sequence of anti-seizure treatments referred to the order that ASMs or a ketogenic diet were introduced according to the patient's medical advice (prescriptions) at the time of the visit to our hospital. Neurodevelopmental status was determined based on the diagnostic information, particularly at the first and last visit. Intellectual disability, defined as an intellectual quotient (IQ) < 70, and neurodevelopmental delay, defined as developmental quotient (DQ) < 70, were diagnosed based on a formal neuropsychological evaluation, except in cases where formal testing was not possible due to severe cognitive disability. Side effects were determined based on diagnostic information and laboratory test results.

### Statistics

Statistical analysis was performed using SPSS 22.0. Descriptive statistics were used for a patients' gender, age, number of visits, and description of comorbidities. The chi-square test was used to analyze the associations of factors with seizure freedom outcome. *P* ≤ 0.05 was considered statistically significant.

## Results

### General information

One hundred and seventy-seven patients (101 males and 76 females) with infantile spasms were included in this study. The total number of visits per patient was 53.73 ± 26.40, ranging from three to 203, with a median of 47 visits, totaling 9,511. Age at the initiation of ketogenic diet therapy was 26.61 ± 17.25 months, ranging from 1.67 to 133.57 months, with a median of 17.73 months. Other demographic characteristics are shown in Table 1.

**Table 1:** Demographic characteristics in infantile spasms treated with a ketogenic diet.

Variables	Mean ± SD	Range	Median	Number
Age at first visit to our hospital (months)	13.45 ± 11.33	0 to 89	8	177
Age at the last visit (months)	70.21 ± 22.60	27.17 to 181	63.20	177
Timespan from the first to the last visit (months)	56.35 ± 17.34	24.23 to 103.5	53.27	177
Total number of visits	53.73 ± 26.40	3 to 203	47	177
Age at the initiation of a Ketogenic (months)	26.61 ± 17.25	1.67 to 133.57	17.73	177
Duration of ketogenic diet (days)	374.03 ± 302.57	9 to 1989	205	99

The age at the initiation of ketogenic diet therapy was 26.61 ± 17.25 months, ranging from 1.67 to 133.57 months, with a median of 17.73 months. The average duration of ketogenic diet therapy was 374.03 ± 302.57 days, ranging from 9 to 1989 days, with a median of 205 (*n* = 99) days.

Seizure reduction by ketogenic diet therapy at different timepoints is shown in Table 2. After initiation at 3, 6, 12, and 24 months, the responder rate (defined as seizure reduction of more than 50%) was 54%, 47%, 27%, and 16% (intention to treat), respectively. Accordingly, the rate of seizure freedom was 17%, 17%, 14%, and 10% at different timepoints. The peripheral blood level of beta-hydroxybutyrate (BHB) and glucose in seizure-free patients is listed in Table 2.

**Table 2:** Responder rate of infantile spasms with classical ketogenic diet therapy.

Variables	At 3 months	At 6 months	At 12 months	At 24 months
Seizure reduction >50%, %	54	47	27	16
Seizure freedom,%	17	17	14	10
Blood BHB,mmol/l	3.6 ± 0.9	3.7 ± 0.9	3.1 ± 1.1	3.3 ± 0.9
Blood Glucose,mmol/l	4.7 ± 0.7	4.6 ± 0.7	4.6 ± 0.6	4.5 ± 0.8

Outcome of seizure control treated with intention to treat.

### Seizure freedom effect

**Figure 1:**
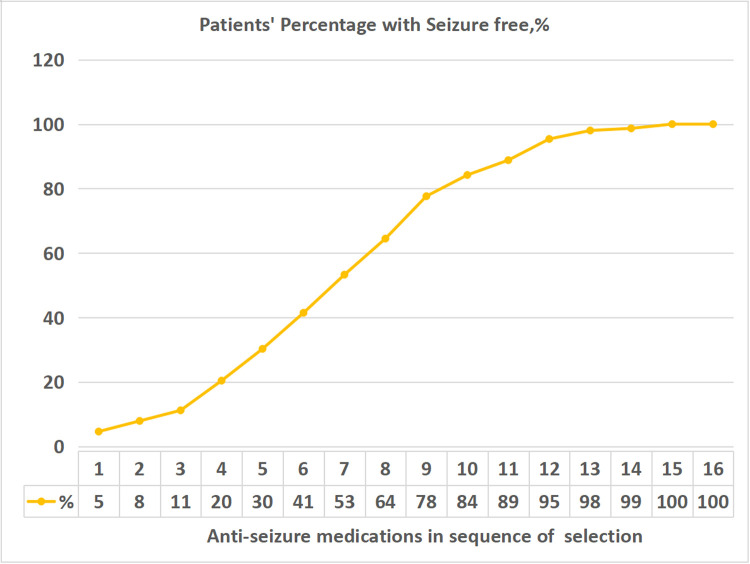
Cumulative percentage of seizure freedom in the order of when a ketogenic diet was introduced.

A total of 152 out of 177 (86%) patients achieved seizure freedom before the last visit. When the cumulative rate of seizure freedom was plotted in terms of the order of when ketogenic diet therapy was introduced as a treatment, it was possible to see that the seizure freedom rate began to increase in patients using a ketogenic diet as the fourth choice. The seizure freedom rate continued to increase until the ketogenic diet was the 11th choice for use in patients. From the 12th choice onwards, the increase in the seizure freedom rate gradually diminished. The seizure freedom rate did not differ significantly by the initiation order of ketogenic diet therapy (*P* > 0.05) (Table 3, Figure 1).

**Table 3:** Rates of seizure freedom in the order of when a ketogenic diet was introduced (*N* = 177).

KD selection order	1	2	3	4	5	6	7	8	9	10	11	12	13	14	15	16
Patients with seizure freedom	7	12	17	31	46	63	81	98	118	128	135	145	149	150	152	152
Patient total number	8	14	22	38	56	78	98	117	139	152	160	170	174	175	177	177
Cummulative rate of seizure freedom (%)	5	8	11	20	30	41	53	64	78	84	89	95	98	99	100	100

KD, ketogenic diet. The order of ketogenic diet therapy meant it was selected as an anti-seizure treatment.

### The use of anti-seizure medications (ASMs)

**Figure 2:**
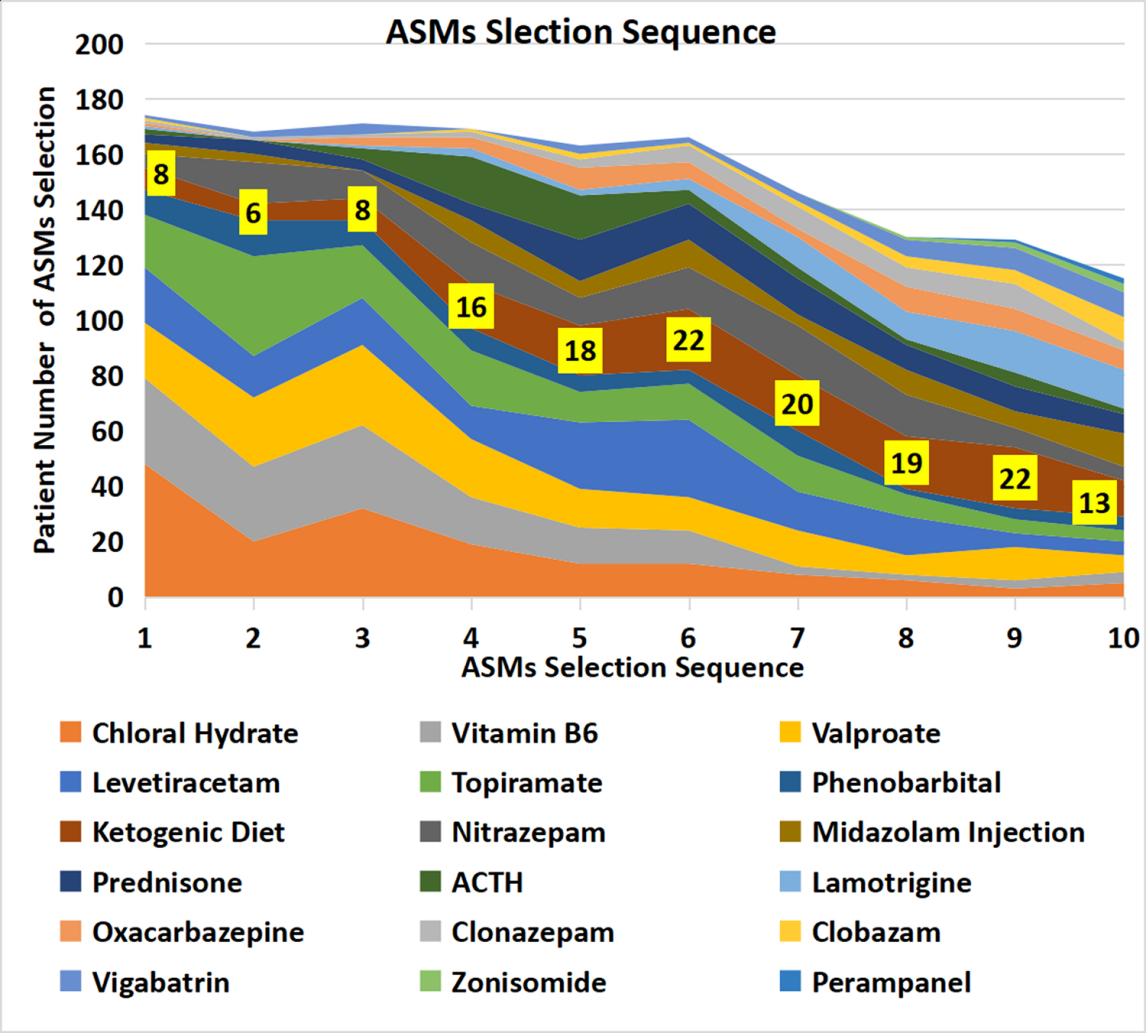
Patient numbers according to anti-seizure treatment selection sequence. The strip denoted with patient numbers (yellow background) represents the ketogenic diet. The width of the strip represents the number of patients who selected the ASM.

By contrast to standard treatment, the top five drugs used as the first-choice treatment in this cohort were chloral hydrate solution, vitamin B6 injection, valproate, levetiracetam, and topiramate. The second choice ASMs in this cohort included the above five drugs and nitrazepam. Ketogenic diet therapy began to be used increasingly from the fourth choice until the 11th choice (Table 4, Figure 2).

**Table 4:** Patient numbers according to the order of anti-seizure treatment selection.

Order of ASM selection	1	2	3	4	5	6	7	8	9	10
Chloral hydrate	48	20	32	19	12	12	8	6	3	5
Vitamin B6	31	27	30	17	13	12	3	2	3	4
Valproate	20	25	29	21	14	12	13	7	12	6
Levetiracetam	20	15	17	12	24	28	14	14	5	5
Topiramate	19	36	19	20	11	13	13	8	5	4
Phenobarbital	9	13	9	8	6	5	9	2	4	5
Ketogenic diet	8	6	8	16	18	22	20	19	22	13
Nitrozepam	5	15	10	15	10	15	18	15	7	5
Midazolam	4	3	0	8	6	10	4	9	6	12
Prednisone	3	5	4	6	15	13	13	9	9	7
Corticotrophin	2	0	4	17	16	5	4	2	5	2
Lamotrigine	1	0	1	3	2	4	11	10	15	14
Oxcarbazepine	1	0	3	4	8	6	3	9	8	7
Clonazepam	1	1	1	2	3	6	8	7	9	3
Clobazam	1	0	0	1	2	1	2	4	5	9
Vigabatrin	1	2	4	0	3	2	3	6	8	9
Zonisamide	0	0	0	0	0	0	0	1	2	3
Perampanel	0	0	0	0	0	0	0	0	1	2

### Choice of anti-seizure treatment in three different time periods

**Figure 3:**
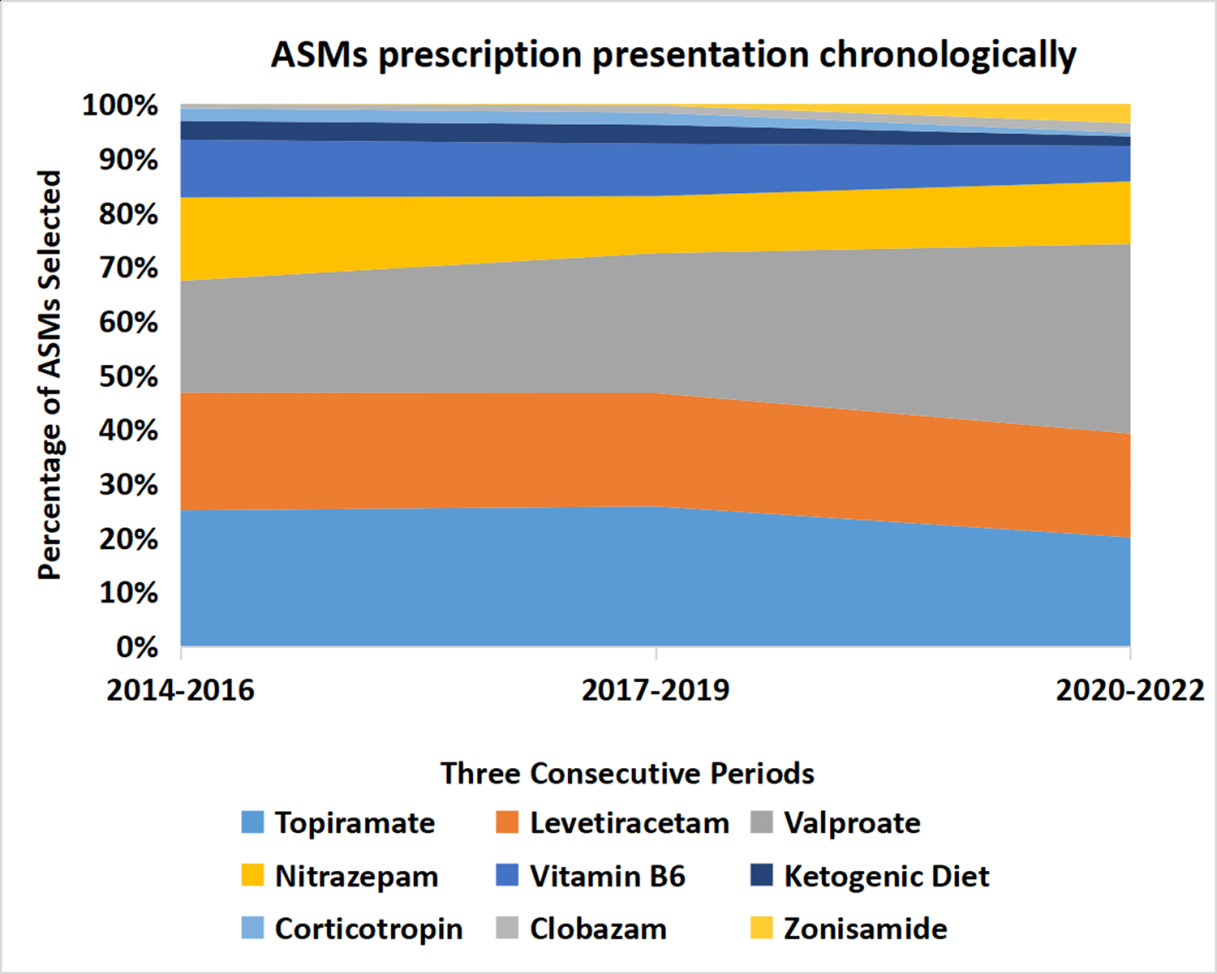
The percentage of ASM prescriptions did not change significantly over three time periods: 2014–2016, 2017–2019, and 2020–2022. ASMs, anti-seizure medications (including ketogenic diet). The width of each strip represents the percentage of ASM prescriptions.

When dividing the study duration into three time periods, 2014–2016, 2017–2019, and 2020–2022, we found a small insignificant change in the choice of ASMs over time. Consistently among the three periods, the top five choices of ASMs were topiramate, levetiracetam, valproic acid, nitrazepam, and vitamin B6, while corticotropin, vigabatrin, and ketogenic diet were not in common use (Table 5, Figure 3).

**Table 5:** Percentage of anti-seizure treatment prescriptions that did not change significantly in three time periods.

% of ASMs selected	2014–2016	2017–2019	2020–2022
Topiramate	17.32	17.94	11.51
Levetiracetam	15.05	14.58	10.99
Valproate	14.27	17.96	20.10
Nitrazepam	10.65	7.34	6.62
Vitamin B6	7.36	6.73	3.75
Chloral hydrate	6.69	6.00	5.14
Midazolam	6.29	3.35	5.34
Phenobarbital	4.88	2.30	2.40
Lamotrigine	2.86	4.88	8.36
Prednisone	2.82	3.86	1.63
Ketogenic diet	2.42	2.44	1.03
Oxcarbazepine	1.96	4.87	6.39
Corticotropin	1.60	1.55	0.36
Clonazepam	1.49	2.70	4.35
Clobazam	0.55	0.95	1.03
Zonisamide	0.00	0.17	2.04

### Developmental Status and disease progression at the last hospital visit

At the last visit (a median of 63.20 months after the first visit), there were significant increases in the proportions of patients having a diagnosis of drug-resistant epilepsy (3.39% vs. 44.89%), neurodevelopmental delay (31.07% vs. 68.75%), generalized epilepsy (48.02% vs. 79.55%), and developmental epileptic encephalopathy (1.13% vs. 9.09%). In addition, the proportions of patients with movement disorders (27.68% vs. 26.70%), focal epilepsy (27.12% vs. 30.68%), and infectious diseases (22.03% vs. 26.14%) remained high, although not significantly compared with the first visit. A total of 110 out of 175 (63%) patients were still on ASMs at the last visit, 60 out of 175 (34%) were receiving ketogenic diet therapy, and 114 out of 175 (65%) continued with anti-seizure treatment. These data suggest a poor long-term outcome (Table 6).

**Table 6:** Changes in diagnosis at the last inpatient visit.

Diagnosis	First admission	Rate of diagnosed	Last admission	Rate of diagnosed	*P* value
Patient number	177		176		
Neurodevelopment delay	55	31.07	121	68.75	<0.0001
Movement disorder	49	27.68	47	26.70	0.8757
Hearing disorder	2	1.13	1	0.57	0.7736
With focal seizures	4	2.26	4	2.27	0.7736
With focal epilepsy	48	27.12	54	30.68	0.5833
Autism spectrum disorder	2	1.13	1	0.57	0.9930
Encephalomalacia	3	1.69	4	2.27	0.9930
Developmental epileptic encephalopathy	2	1.13	16	9.09	<0.0001
Drug-resistant epilepsy	6	3.39	79	44.89	<0.0001
With encephalitis	7	3.95	4	2.27	0.3681
Infections	39	22.03	46	26.14	0.4807
Generalized epilepsy	85	48.02	140	79.55	0.0036
Brain atrophy	1	0.56	2	1.14	0.7776

### Adverse events of anti-seizure treatment

Adverse events included malnutrition, defined as nutrient deficiency or an excess of a particular nutrient (41, 23%), diarrhea (40, 23%), anemia (21, 12%), constipation (22, 12%), vomiting (17, 10%), hypoalbuminemia (7, 4%), myocardial injury (7, 4%), urinary calculi (5, 3%), gallstone (3, 2%), and abdominal pain (4, 2%). There were no patient deaths during the study period.

## Discussion

In this study, we retrospectively analyzed the long-term outcomes of infantile spasms in a single-center cohort of patients treated with ketogenic diet therapy, based on the hospital electronic medical record system. Patients did not receive standard first-line treatment (hormone and/vigabatrin) but instead were treated with ordinary anti-seizure medications in combination with ketogenic diet therapy. All patients had medical records for more than 2 years, and the median time span was 53.27 months. Ketogenic diet therapy was used increasingly from its selection as the fourth choice of treatment until the 11th choice of treatment. The median duration of ketogenic diet therapy was 205 days. The long-term retention rate of KD was 34% (median time, 53.27 months), similar to that of a systematic review and meta-analysis on KD in infants, which was 27% at 24 months (16). Our spasm-free rate at 24 months was 10%, similar to 9.54% at 12 and 24 months (median, range 8.69%–32.69%) (17). Recommended first-line agents (18–20), such as corticotropin and vigabatrin for infantile spasms, were not commonly used in this cohort; only a small proportion of patients used them as the first to third choice of treatment. Although the rate of spasm freedom was high (86%) at the last visit, this was not the case in the short term. In addition, the proportions of patients with neurodevelopmental delay, generalized epilepsy, developmental epileptic encephalopathy, and drug-resistant epilepsy were higher than those at the first visit. These results indicate unfavorable outcomes in these patients in terms of development, cognition, and epilepsy control after 4 years of medical visits and treatments, with 69% of patients displaying neurodevelopmental delay.

The outcomes regarding seizure control and neurodevelopment in our retrospective study are similar to other studies, including a prospective multicenter study. A previous study [International Collaborative Infantile Spasms Study (ICISS)] demonstrated that, in patients receiving a combination of standard first-line vigabatrin and glucocorticoids, and those receiving glucocorticoids alone, 30.0% and 29.2% of them displayed seizures, respectively, while 15.0% and 15.7% of them showed epileptic spasms at 18 months; the Vineland Adaptive Behaviour Scales (VABS) scores were 73.9 and 72.7, respectively. In addition, the two groups had similar serious adverse effects (21, 22). The United Kingdom Infantile Spasms Study (UKISS) (23) showed that, at 14 months, the seizure freedom rate of epileptic spasms was 75% and 76% in the hormone and vigabatrin groups, respectively, and VABS scores averaged 78.6 and 77.5, which were not significantly different. In infants with no identified etiology, the mean VABS score [SD] was higher in those receiving hormone treatment than in those receiving vigabatrin (88.2 [17.3] vs. 78.9 [14.3]; difference 9.3, 95% CI: 1.2–17.3; *P* = 0.025). In a follow-up of 77 patients of a mean age of 4 years, the medians of VABS scores with hormone therapy and vigabatrin therapy were 60 and 50, respectively (*P* = 0.091), and developmental status and epilepsy outcomes were similar between the two groups, with 17/39 (44%) and 20/37 (54%) patients still having seizures, respectively (*P* = 0.5921). Five patients in each group presented with epileptic spasms (13% vs. 14%). However, those with a proven etiology were more likely to have continued seizures than those with no identified etiology [24/39 (62%) vs. 13/37 (35%): chi-squared = 5.3; *P* = 0.021] (24). Therefore, even with standard treatment, the long-term outcomes on development and seizure control in patients with infantile spasms remain to be improved. In addition, KD had both an anti-seizure and neuroprotective effect. Further prospective multicenter controlled studies on the efficacy of standard treatment and non-standard treatment combined with ketogenic diet therapy are warranted.

Corticotropin and vigabatrin are recommended therapies for infantile spasms; in particular, vigabatrin is used as the first option for tuberous sclerosis complex with infantile spasms (19, 20). Owing to the limited supply of the two drugs in mainland of China, most of the first-line drugs used are general ASMs, such as topiramate, valproate, levetiracetam, and vitamin B6. These alternative options may potentially result in significantly impaired cognitive development because if epileptic spasms are not controlled early then the brain will suffer continuous injury. Corticotropin is a natural hormone extracted from the pituitary gland of animals in mainland China. It is not widely used in clinics due to its low production caused by complicated processes and marketing factors, and patients' fear of the side effects of the drug. Vigabatrin entered the market in mainland China in 2022, so its accessibility is not guaranteed. These reasons contributed to the reduced use of the two first-line agents. Glucocorticoids, including prednisone, prednisolone, and methylprednisolone, are cheaper and more easily available. A study by Zhong J et al. found that prednisone treatment at 4 mg/kg of body weight per day for 2 weeks followed by tapering is effective at treating infantile spasms and that 16 out of 20 (80%) patients experienced a termination of spasms (25). Another oral prednisone therapy in a larger sample size confirmed that there was no significant difference in the rate of short-term spasm freedom between higher (4 mg/kg) and conventional (2 mg/kg) dose groups (56% and 52%, respectively) (*n *= 54) (26). A study by Kossoff et al. produced similar results, with 10 out of 15 (67%) patients achieving seizure freedom after 2 weeks of high-dose oral prednisolone (40–60 mg/day) (27, 28). Since 2006, the authors have used high-dose oral prednisolone instead of injectable corticotropins for the treatment of infantile spasms (28). Zhisheng Liu and other researchers confirmed that the short-term use of oral methylprednisolone, intramuscular injection of corticotropin, and oral prednisone have similar effects on short-term seizure freedom rates (electro-clinical), which were 53.7%, 60.75%, and 51.43%, respectively. Oral methylprednisolone exerts efficacy more rapidly than the other two (29, 30). UKISS (23, 24) has confirmed that the neurodevelopment of children with infantile spasms of unknown etiology receiving hormones is better at the ages of 14 months and 4 years. Therefore, oral methylprednisolone or prednisone may be used as an alternative in regions with poor availability of corticotropin injections and limited resources. The early use of combined hormone and vigabatrin may be more effective at controlling epileptic seizures but there are no significant differences in the long-term outcomes for development and seizure control (21, 23). Similar results have been reported in other studies, suggesting that the currently recommended protocol for infantile spasms, which is not ideal in terms of long-term outcome, still requires more research in terms of pathogenesis and the exploration of better treatment options. The pooled ratio (2,967 patients) for a good neurodevelopmental outcome was 0.236 (95% CI: 0.193–0.286). The ratio for a good neurodevelopmental outcome was higher for cryptogenic infantile spasms [0.543 (95% CI: 0.458–0.625)] than for symptomatic infantile spasms [0.125 (95% CI: 0.09–0.171)] [*P* < 0.001]). The risk ratio for a good neurodevelopmental outcome in patients with a lead time to treatment of <4 weeks relative to patients with a lead time to treatment of >4 weeks was 1.519 (95% CI: 1.064–2.169) (31). The neurodevelopmental outcome has, overall, been poor in patients with infantile spasms since the publication of the first guideline on infantile spasms. Although cryptogenic infantile spasms have a better prognosis than symptomatic infantile spasms, the outcome remains poor.

Kossoff et al. (32) reported that, when used as a first-choice treatment in infantile spasms, ketogenic diet therapy offers higher seizure freedom rates, fewer side effects, and lower relapse rates; however, the sample size was small in this study, perhaps due to an insufficient and incorrect recognition of the ketogenic diet and a fear of the initial complicated protocol. Ketogenic diet therapy is not widely used in many regions. Hong et al. (33) reported that the ketogenic diet, when used as the first choice for infantile spasms, resulted in a seizure freedom rate of 10/18 (56%) within 2 weeks of initiation. Epileptic spasms (including both newly diagnosed and not newly diagnosed patients) were terminated in 37% of patients within a median of 2.4 months after the initiation of a ketogenic diet, and the freedom lasted for at least 6 months. In addition, 62% of epilepsy patients showed an improvement in development. When a ketogenic diet is initiated before 1 year of age, the previous use of ≤3 anti-seizure medications is an independent predictor of >90% seizure reduction 12 months after initiation. A long-term follow-up (at least for a median of 2.5 years) (34) showed that the seizure-freedom rates in the ketogenic diet and corticotropin groups were 40% and 27%, respectively (*P* = 0.18), and the proportions of patients with normal neurodevelopment were 23% and 15%, respectively (*P* = 0.38). However, the long-term seizure freedom rates were similar for patients with no previous use of vigabatrin (33% vs. 35%, *P* = 0.90), and the rates of age-appropriate development were 30% vs. 15%, respectively (*P* = 0.30). Therefore, in the future, large sample multicenter clinical trials should be organized as early as possible to evaluate the long-term efficacy of combined oral first-line drugs (glucocorticosteroids and vigabatrin) or ketogenic diet therapy as a first-line treatment for infantile spasms. After failure of the first treatment, subsequent treatments should all be scheduled within 2 weeks (5, 35) to achieve seizure freedom as soon as possible and improve the long-term outcome for cognition.

One limitation of this real-world study was that it was a retrospective study based on hospital medical records. The reliability of the data needs to be improved, including seizure frequency recordings and the use of diagnostic information to represent developmental assessment. Some patients have been diagnosed and treated in other hospitals, which may also lead to incomplete data. In addition, there was no clear distinction between newly diagnosed patients and patients with drug-resistant epilepsy.

In conclusion, in this real-world study, we found that in a resource-limited region with a lack of first-line medications and poor development of ketogenic diet therapy, the drugs of choice for infantile spasms are generally not the recommended steroids or vigabatrin, but topiramate, levetiracetam, valproic acid, nitrazepam, and vitamin B6. The use of ketogenic diet treatment was increased from the fourth treatment choice. Efforts should be made to improve the use of first-line anti-seizure medications, alone or in combination with the early initiation of ketogenic diet therapy, to achieve seizure freedom as early as possible, thereby improving neurodevelopment and outcomes in general. Prospective multicenter clinical trials to evaluate the long-term efficacy and safety of ketogenic diet therapy as a first-line treatment should also be conducted to further investigate the pathogenesis and find new therapeutic avenues.

## Data Availability

The raw data supporting the conclusions of this article will be made available by the authors, without undue reservation.
